# DNA methylation markers in the detection of hepatocellular carcinoma

**DOI:** 10.1016/j.ejca.2023.112960

**Published:** 2023-06-28

**Authors:** Siyu Fu, José D. Debes, André Boonstra

**Affiliations:** a Erasmus MC University Medical Center, Department of Gastroenterology and Hepatology, Rotterdam, the Netherlands; b Department of Medicine, University of Minnesota, Minneapolis, MN, USA

**Keywords:** Hepatocellular carcinoma, DNA methylation markers, Liquid biopsy, Aetiology, Early-stage HCC

## Abstract

Hepatocellular carcinoma (HCC) is the most common primary liver malignancy and has a poor prognosis. Epigenetic modification has been shown to be deregulated during HCC development by dramatically impacting the differentiation, proliferation, and function of cells. One important epigenetic modification is DNA methylation during which methyl groups are added to cytosines without changing the DNA sequence itself. Studies found that methylated DNA markers can be specific for detection of HCC. On the basis of these findings, the utility of methylated DNA markers as novel biomarkers for early-stage HCC has been measured in blood, and indeed superior sensitivity and specificity have been found in several studies when compared to current surveillance methods. However, a variety of factors currently limit the immediate application of these exciting biomarkers. In this review, we provide a detailed rationalisation of the approach and basis for the use of methylation biomarkers for HCC detection and summarise recent studies on methylated DNA markers in HCC focusing on the importance of the aetiological cause of liver disease in the mechanisms leading to cancer.

## 1. Introduction

Liver cancer is one of the most prevalent human tumours and contributes significantly to the global cancer burden, with an estimated 810,000 deaths per year [1]. Over 80% of all liver cancers are classified as hepatocellular carcinoma (HCC). HCC typically arises in the background of cirrhosis, although up to 20% of cases can develop in non-cirrhotic livers [2]. Infections with the hepatitis B virus (HBV) or hepatitis C virus (HCV) are major causes of HCC worldwide. Although two large clinical cohorts showed that with the advent of new therapies, the incidences of HBV- and HCV-related HCC have significantly reduced [3,4], the lifetime risks of HCC in patients with chronic HBV and HCV are still high [1,5,6], especially in those who have been diagnosed with cirrhosis [7]. In recent years, the rapidly rising incidence of metabolic syndrome in many countries has shifted the aetiology of HCC away from viral hepatitis towards non-alcoholic fatty liver disease (NAFLD) [8]. In addition, the number of patients with alcoholic liver disease (ALD)-related HCC has also increased across continents and cirrhosis secondary to alcohol intake is the dominant factor in the development of HCC in many parts of the world, including Central and Eastern Europe [9]. Other potential risk factors that contribute to hepatocarcinogenesis include aflatoxin B1, tobacco, and rare genetic mutations [10]. The stratification of HCC is largely based on the Barcelona Clinic Liver Cancer (BCLC) scoring system. Early-stage HCC is usually referred to BCLC 0/A stage [11]. The prognosis of HCC is driven by tumour stage, with curative options, patients have a more than 70% survival rate for early-stage HCC, while for symptomatic advanced-stage cases treated with systemic therapies, the survival rate only achieved 1–1.5 years [12,13]. Hence improved identification of early-stage HCC and prediction of those who are going to develop HCC is urgently needed. Currently, the surveillance of HCC in clinical practice relies on ultrasonography and alfa-fetoprotein (AFP). Given the poor sensitivity of AFP when used alone in surveillance of HCC (41–65%) [14], ultrasonography is recommended by most hepatological societies (European Association for the Study of the Liver (EASL), The American Association for the Study of Liver Diseases (AASLD), and The Asian Pacific Association for the Study of the Liver (APASL)) with or without AFP. However, the sensitivity of ultrasonography for detecting early-stage HCC is only 47%, and when combined with AFP, this increases to just 63% [15]. For this reason, the search for more sensitive blood biomarkers to detect early-stage HCC is an active field of research. Indeed, a large number of candidate markers are currently being studied, including protein markers (i.e. glypican-3, Golgi protein 73, AKR1B10), inflammatory markers (interleukins and angiogenic factors), and microRNAs (i.e. microRNA-122 and microRNA-21) [16]. Some of these markers are combined in algorithms consisting of gender, age, AFP-L3, AFP, and Des-Carboxy-Prothrombin (DCP), called the GALAD score, which seems to have a better profile in predicting the probability of developing HCC in patients with chronic liver disease, but has not been validated across the globe [17].

Other candidate biomarkers for the early detection of HCC are specific methylated genes detected in cell-free DNA (cfDNA) isolated from blood (Fig. 1). In general, cfDNA is derived from dead cells, such as leucocytes, but in patients with cancer, a fraction can also be released by the tumour itself into the circulation as a result of necrosis or apoptosis of tumour cells. This circulating tumour-derived DNA (ctDNA) can be analysed for methylation patterns of genes that are specific for the tumour, and not found in healthy cells. This approach holds great promise because of the advances in the field to determine DNA methylation from cfDNA by highly sensitive techniques in a minimally invasive manner using plasma or serum [18]. Indeed, a comparison of genome-wide profiles in cfDNA has revealed hypermethylated or hypomethylated genes that were reported to be specific for a tumour [19], and, therefore, potentially discriminative between early-stage HCC and cirrhosis [20]. Exploration of the details of these markers, referred to as DNA methylation markers (DMMs) in the detection of early-stage HCC and the heterogeneity between different liver diseases may be applicable and highly relevant for the surveillance of HCC. In this review, we provide an exhaustive description of these markers as well as their differences across variable causes of HCC.

## 2. Mechanisms of DNA methylation and role in tumourigenesis

Genomic DNA can be modified, resulting in modulation of transcriptional regulation. This process is called epigenetic modification, and includes methylation of specific nucleobases in DNA. During DNA methylation, a methyl group provided by S-adenosylmethionine is covalently coupled to DNA on the fifth position of cytosine (5-mC) [21,22]. This cytosine methylation reaction is mediated by various DNA methyltransferases (DNMT): DNMT1, DNMT3a, and DNMT3b are involved in inducing or maintaining global cytosine methylation [23]. Under normal circumstances, DNA methylation is observed on cytosine-guanine dinucleotides (CpGs) dispersed randomly throughout the DNA sequence, and the promotor and first exon regions are virtually unmethylated [24,25]. In contrast, in cancerous cells, this is the opposite, with relatively high methylation rates observed in the promotor and first exon regions (Fig. 2) [25].

DNA methylation at the promoter regions has major consequences since it modulates transcriptional regulation leading to changes in gene expression. How DNA methylation contributes to inhibit gene expression still remains unclear. Studies have shown that DNA methylation generates a physical barrier for some transcriptional factors, thereby preventing access to promoter-binding sites [23]. DNA methylation has also been shown to induce gene suppression by a mechanism associated with methyl-CpG-binding domain proteins. After the interaction of these domain proteins with methylated CpGs, repressive epigenetic modification enzymes are recruited, resulting in chromatin condensation and gene silencing [26–28]. In general, DNA methylation of the gene promoter is regarded as an initiation step for establishing an inactive transcriptional status. However, some studies recently described that DNA methylation at the promotor site does not always lead to gene suppression. For example, a differentially methylated region containing 34 CpGs was identified in the distal part of the telomerase reverse transcriptase (TERT) promoter, and hypermethylation of this region was associated with increased TERT expression [29].

In cancer, except for the hypermethylation of CpG islands in the transcriptional regulatory region, the genome of the tumour is characterised by an overall tumour-wide DNA hypomethylation, including hypomethylation of repetitive DNA sequences and demethylation of coding regions and introns. 5-mC can be actively reversed to unmodified cytosine through ten-eleven translocation (TET)-dioxygenase-mediated oxidation to 5-hydroxymethylcytosine (5-hmC) [30]. During tumourigenesis from benign cellular proliferation to invasive tumour cell growth, the degree of hypomethylation of genomic DNA increases [31]. As a result, there is a greater chance of undesired mitotic recombination. Transposable elements are then reactivated and can integrate at random sites in the genome, leading to mutagenesis and genomic instability [23,32–34]. In addition, studies have also found that changes in DNA methylation may lead to the development of mutations; and mutation rates tend to be higher at methylated CpGs than at non-methylated ones [35,36]. Hence, the frequencies of DNA hypermethylation and hypomethylation at certain regions are thought to increase the rate of tumourigenesis and progression of cancer [37,38], suggesting that the DNA methylation status of genes can act as predictive markers for cancer development and progression. It is within this mechanistically context that DMMs, as specific methylation sites within cancer are known, have shown significant promise and interest as a surveillance tool for HCC.

## 3. Techniques for the detection of DNA methylation

The methylation of DNA within certain genes cannot be determined by regular quantitative real-time polymerase chain reaction (qPCR) since the distinction between cytosine and methylated cytosine requires additional technical modifications. Therefore, approaches have been developed to discriminate between methylated and non-methylated cytosine. One such approach is based on bisulfite conversion of cytosine. During this chemical reaction, sodium bisulfite converts normal cytosine residues to uracil residues in DNA, while leaving methylated cytosine intact [39]. Following bisulfite treatment, the two converted strands of the DNA template are no longer complementary [40]. One major drawback of the method of bisulfite conversion is that high amounts of input DNA are lost due to degradation (sometimes up to 90%), which complicates the assay when the sample quantity is limited. Based on the bisulfite-converted sequences, several techniques were developed to determine the methylation status of genes, including whole genome bisulfite sequencing, reduced representative bisulfite sequencing, pyrosequencing, quantitative methylation-specific PCR, and microarray-based techniques [41]. A summary of these techniques, their requirements with respect to input DNA needed, and assay characteristics are presented in Table 1. Whole genome bisulfite sequencing is the gold standard for genome-wide measurements of DNA methylation levels. It can detect the methylation state of nearly every CpG site [42–44], but it is expensive and requires deep sequencing [41]. Compared to whole genome bisulfite sequencing, reduced representative bisulfite sequencing (RRBS) is relatively cost-effective since it targets CpG-rich regions while ignoring CpG-poor regions [42,45,46]. Recently, Van Paemel et al. reported on the application of RRBS on cfDNA with minute DNA input [47,48]. Pyrosequencing, on the other hand, is used for quantitative DNA methylation analysis and is suitable for both CpG-poor and CpG-rich regions. The main disadvantage of this method is that only relatively short regions (less than 350 bp) can be measured [49]. This can, however, be tackled by using more sequencing primers on one amplicon or by performing serial pyrosequencing [50]. Quantitative methylation-specific PCR is a PCR-based method that uses primers designed specifically for methylated and unmethylated alleles of a chosen region. This method is suitable for the evaluation of the methylation status of small numbers of specific genes, is relatively cheap, and data analysis is straightforward. However, standardisation of the PCR-based assays, including the design of optimal methylation-specific primers and probes, is important to avoid false positive or false negative results [50]. Microarray-based techniques can be used after the digestion of DNA with methylation-specific enzymes or bisulfite conversion [51]. One of the most used array-based platforms is the Illumina Infinium Human Methylation 450 Bead Chip array (Illumina HM450), which covers more than 485,000 CpG dinucleotides in 99% of known genes and 96% of CpG islands at a single base resolution [43,52,53]. This array has been used as a reference platform for DNA methylation in The Cancer Genome Atlas Consortium (TCGA), but it does require large amounts of DNA [54].

Other methods that do not require the bisulfite conversion step are also available. Some methods use methylation-specific enzymatic digestion of DNA [55,56], such as the recently described methylated DNA sequencing (MeD-seq), which uses LpnPI endonuclease, a novel methylation-dependent enzyme that specifically cuts 16 bp upstream and downstream from methylated CpG sites to generate 32 bp fragments [57,58]. Also, TET-assisted pyridine borane sequencing (TAPS) does not require bisulfite conversion. The method converts methylated cytosines into dihydrouracil and subsequently to thymine, and requires low input DNA requirement (about 1 ng DNA), and a high sensitivity and specificity have been reported [59,60]. Thus, these methods allow for genome-wide methylation profiling without the need for harsh bisulfite treatment. Also, enrichment-based techniques, such as methyl-DNA immunoprecipitation (MeDIP) and methyl-binding domain capture (MBDCap), do not need bisulfite conversion. MeDIP is based on the immunoprecipitation of single-stranded molecules containing methylated CpGs by using a monoclonal antibody specifically against 5-mC, while MBDCap captures double-stranded methylated DNA fragments by MBD-based proteins [61]. Both of them can be combined with next-generation sequencing [62–64]. These techniques are rather recent and novel, and further validation and standardisation are necessary before broad use.

## 4. DNA methylation as a potential marker for cancer detection

Determination of the methylation status of a particular tumour type is usually performed by first evaluating genomic DNA from tumour biopsies or resections since relatively large amounts of tumour-derived DNA can be isolated. This approach will first provide candidate markers specific to the tumour. However, when the methylation status is intended to be assessed for screening purposes to detect HCC at an early stage this detection should be done in peripheral blood which is more reasonable as a screening technique. Therefore, the scientific community has attempted to identify DNA in blood that resembles that of a tumour. This is often referred to as a ‘liquid biopsy’. Recently, Guo et al. and Moss et al. found that cfDNA in blood of patients reflects tumour tissue contributions that are in line with clinical findings [65,66]. Moreover, it has been demonstrated for various cancer types that ctDNA in the blood can be used in methylation studies for early-stage HCC. This ctDNA, is a highly variable fraction of cfDNA, ranging from 0.01% to more than 50% [67]. The major challenge for this is how to identify the small amounts of ctDNA out of total cell-free DNA in the blood. For the detection of ctDNA assays, the use of plasma is thought to be better than serum as the latter contains higher levels of genomic DNA generated mostly from leucocyte lysis that occurs during the clotting process when isolating serum [68]. In order to acquire enough ctDNA for analysis, a relatively high volume of plasma is needed [57]. Also, sensitive techniques, such as digital droplet PCR, can be applied, but the drawback of this assay is the low capability of multiplexing (identifying multiple targets at once). Indeed, current approaches require the identification of multiple DMMs for cancer detection, and it is, therefore, necessary to assess multiple methylation sites for better precision in detecting a tumour. Similarly, targeted sequencing-related techniques can allow the interrogation of multiple loci with high sensitivity through the use of methods that suppress background noise [69]. Therefore, ctDNA has been widely used in DNA methylation studies and showed encouraging results in the early detection of tumours, such as HCC [70–72], lung cancer [73], and colorectal cancer [74]. There are many advantages of using ctDNA from blood samples instead of relying on tumour tissue, but the most important one is the non-invasiveness of sample collection, which causes little discomfort for patients, and thus allows the possibility for more frequent screening using methylated DNA as blood biomarkers.

## 5. DNA methylation in HCC

### 5.1. DNA methylation markers in the diagnosis of early-stage HCC

Early detection is critical for HCC since more treatment options are available with clear beneficial effects on survival rates [75]. Xu et al. started the comparison of 401 DMMs generated by paired liver tissue DNA and plasma cfDNA from the same patients (n = 28) and subsequently validated this in cfDNA (n = 1933). The selected 10 DMMs yielded more than 80% sensitivity and 90% specificity for HCC (n = 1098) compared to healthy individuals (n = 835) in the following cohort. Also, these 10 DMMs showed a high area under the curve (AUC) of 0.97 in the HCC cohort and could differentiate patients with HCC from patients with other liver diseases (HBV, HCV, and NAFLD) [70]. A different study sequenced DNA from cirrhotic livers and HCC and identified different DMMs: Kisiel et al. identified a number of different DMMs, which were later validated on cfDNA from plasma of HCC patients (n = 95, 48% early-stage), cirrhosis patients and healthy individuals [20]. The study defined a six-marker DMMs panel consisting of HOXA1, EMX1, AK055957, ECE1, PFKP and CLEC11A normalised by B3GALT6, which showed a 95% overall HCC sensitivity and 92% specificity in detecting HCC. Importantly, this panel also detected more than 90% of early-stage HCC (n = 46, BCLC 0/A). As a follow-up of the study, 4 of the identified DMMs, HOXA1, EMX1, TSPYL5 and B3GALT6 were combined with the protein biomarkers AFP and AFP-L3 and tested on cfDNA from 135 HCC cases and 302 controls (87% cirrhosis). The panel achieved a sensitivity of 71% with a 90% specificity for early-stage HCC (n = 76) [76]. Subsequently, the same group improved the performance with only three DMMs (HOXA1, TSPYL5, and B3GALT6) in combination with AFP and gender on cfDNA from plasma, finding an overall 88% sensitivity for HCC detection in 156 cases compared to 245 controls (92% cirrhosis) as well as 82% sensitivity for early-stage HCC (n = 78) [77]. The patient population had diverse underlying liver diseases, with almost 85% ascribed to HCV, NAFLD or alcohol. Another study by Hernandez-Meza et al. demonstrated an increasing proportion of hypermethylated samples from cirrhotic tissue (< 1%), to dysplastic nodules (≥25%), and to early-stage HCC (≥50%) for four DMMs: TSPYL5, KCNA3, SPINT2 and LDHB [78]. Interestingly, TSPYL5 and SPINT2 were also hypermethylated in HCC tissues in the study of Kisiel et al. [20]. In addition, TSPYL5 also showed promising results in distinguishing HCC from paired non-tumour adjacent tissues in the same patients [79,80]. Recently, a multicenter study was performed on cfDNA from patients with HCC (n = 122, 37% early-stage) and controls (37% cirrhosis) using a prespecified diagnostic algorithm consisting of 28 DMMs. This test, known as HelioLiver Test, showed a sensitivity of 76% for HCC detection in patients with early-stage HCC [81]. Currently, a prospective clinical study launched by HelioGenomics on samples from patients with cirrhosis (n = 1600) is ongoing (NCT03694600). Besides DMMs, changes of 5-hmC seem specific to HCC compared to chronic liver disease. Cai et al. performed genome-wide 5-hmC sequencing on cfDNA to develop a 32-gene diagnostic model, which exhibited high AUC (0.85) in the comparison between early-stage HCC and cirrhotic livers as well as chronic HBV livers [82].

To date, methylation of SEPT9 is the first and only blood-based methylation marker approved by the United States Food and Drug Administration (FDA) for colorectal cancer screening [83]. Interestingly, expression of SEPT9 was found to be decreased in HCC via promoter hypermethylation in a study of 304 HCC tissues compared to cirrhotic and normal liver tissues [84]. Also, the SEPT9 hypermethylation status exhibited an AUC of 0.86 in discriminating BCLC-A HCC from cirrhosis using plasma cfDNA in a multicenter study [85]. In line with this, using digital droplet PCR, it was recently demonstrated that the median copy numbers of methylated SEPT9 were higher (6.4 versus 2.0) in patients with HCC (n = 136) compared to chronic liver disease by cfDNA from plasma (Table 2) [86].

Despite the exciting data described above, numerous studies have demonstrated a high degree of heterogeneity among methylation patterns in patients with HCC. One of the most important reasons for this is the differences in major risk factors driving the development of HCC, such as HBV, HCV, alcohol and NAFLD. It has been shown that DNA methylation patterns are different in HCC based on the associated risk factors. Unsupervised hypermethylation clustering on DNA sequenced from 196 HCC tissues compared to adjacent normal tissues exhibited different methylation profiles between HBV- and HCV-related HCC [87]. PAX6 was more frequently hypermethylated in HCC associated to HCV (61%) and less methylated in HBV-related HCC (22%) [88]. Similarly, HCV-related HCC tissues showed higher methylation levels of p16 than HBV-related HCC [89], and methylguanine-methyltransferase (MGMT) was more methylated in alcohol-related HCC compared to non-alcohol-associated HCC [90]. Moreover, differences have been found across infectious and non-infectious causes of HCC. Two studies found that some DMMs are different in NAFLD-related HCC, showing lower methylation levels of TRIM4, PRC1, TUBA1B, WHSC1, MAML3, and higher methylation levels of FLCN and WDR6 compared to viral hepatitis-related HCC [91,92]. In addition to aetiology, age and geographical distribution have also been reported to influence the degree of methylation in HCC. HCC patients over the age of 60 show more hypomethylated LINE-1 in liver tissue compared to younger age groups (30% versus 11%) [90]. Also, HCC patients from Thailand exhibited different methylation levels as compared to HCC patients from France, with higher methylation levels in P14 (4.1 versus 0.7) and DOK1 (30.7 versus 18.1) as well as lower methylation levels in GNMT (8.7 versus 14.8) [90]. All of these speak of the complexities in defining specific DMMs for HCC detection that can encompass multiple HCC causes, and geo-demographic factors in a one-size fits all style.

### 5.2. DNA methylation markers in HBV-related HCC

As mentioned above, the methylation status of specific genes may not be similar between HCCs with distinct aetiological causes. However, only a few studies stratified HCC patients for specific etiologies. For HBV-related HCC, it has been demonstrated that several genes are hypermethylated in HBV-related HCC compared to cirrhosis and healthy individuals.

Lambert et al. performed pyrosequencing on genomic DNA from HBV-positive HCC tissues (n = 32) and found that the promoters of RASSF1A, GSTP1 and DOK1 were significantly hypermethylated with correspondingly decreased mRNA levels as compared to HBV-positive cirrhotic tissues with no HCC (n = 25) [90]. RASSF1A is one of the prototypical tumour-suppressor genes universally inactivated in human malignancies by a methylation-based mechanism [93]. GSTP1 has been suggested to play an important role in protecting cells against damage induced by carcinogens [94]. DOK1 is a member of a family of intracellular adaptor proteins that exhibits tumour-suppressive activity in both hematopoietic and non-hematopoietic malignancies [95]. Using methylation-related PCR techniques, the findings were confirmed for the promoter of RASSF1A and GSTP1 in HCC tissues [93,96]. Another study demonstrated that the methylation ratios of SFRP1 and SFRP5 in HBV-related HCC tissues were higher than in paired non-tumourous tissues [97]. Finally, it has been reported that E-cadherin has a relatively higher methylation ratio in HBsAg-positive HCC tissues compared to HBsAg-negative HCC tissues [98]. E-cadherin has been shown in multiple studies to be a key growth and invasion suppressor in cancer [99].

One of the viral proteins encoded by the HBV virus, the HBV X protein (HBx), has been shown to play a significant role in the development of HBV-related HCC, acting as a carcinogenic protein [100,101]. In HBx-positive HCC tissues, studies found that patients had significantly increased methylation ratios in the promoter of IGFBP-3 [102], and relatively lower mRNA expression of RASSF1A compared to HBx-negative HCC tissues [103]. In HBx-positive HCC cell lines, the activities of different DNMT were also altered [102]. Also, Qiu et al. investigated the expression of RASSF1A in seven cell lines and found that RASSF1A mRNA levels were reduced in the presence of HBx [103]. Similarly, the promoter activity and gene expression of IGFBP-3, GSTP1, and E-cadherin were decreased in HBx-positive cell lines compared to HBx-negative cell lines [102,104,105]. These *in vitro* results in HCC cell lines are in line with the data from HCC tissues, indicating the close correlation among HBV-related proteins, cancer-related genes and methylation in HBV-related HCC.

Only a few studies have specifically studied the differential methylation status in HBV-related HCC using cfDNA isolated from plasma. One study by Zhao et al. compared DMMs in cfDNA of 29 HBV-related HCC with 27 HBV-related cirrhosis, 30 chronic HBV patients and 30 healthy individuals, subsequently validating the results in DNA of 36 HCC tissues [106]. Three genes were identified that distinguished HCC from the other groups: ZNF300 (AUC 0.70), SHISA7 (AUC 0.72) and SLC22A20 (AUC 0.66). The prediction power for HCC of < 0.8 for each methylated gene was good but far from optimal. A study by Hu et al. using methylation-specific PCR on cfDNA observed significantly lower methylation frequencies of UBE2Q1 in HBV-related HCC patients (n = 20) compared to patients with cirrhosis (n = 40) [107]. In combination with different cut-off values of AFP, AUCs between 0.69 and 0.76 were reported in discriminating HCC from cirrhosis and chronic HBV infection. All these studies indicated the potential benefit of DMMs in identifying HCC in HBV-infected patients. However, larger studies addressing the specific status of the virus (immune active disease or controlled viremia) that could affect virus-related methylation rather than cancer-related methylation are needed.

### 5.3. DNA methylation markers in HCV-related HCC

Specific methylation patterns have been observed in patients with HCV-related HCC as compared to other etiologies. A large clinical study looking at HCV-related HCC found dramatic differences in the expression levels of the DNA methyltransferases, with DNMT1, DNMT3A and DNMT3B showing a distinct pattern in patients with HCV genotype 1b and 3a compared to those with genotypes 5 and 6 [108]. Another study showed that the expression of DNMT1, DNMT3A and DNMT3B had an upward trend in blood from healthy individuals compared to chronic HCV infection, HCV-related cirrhosis, and finally to HCV-related HCC, suggesting increased hypermethylation with the progression of the disease stage. Expression of these markers also exhibited over 80% sensitivity in discriminating HCV-related HCC (n = 26) from cirrhosis (n = 45) [109]. Other studies have shown hypermethylation of RASSF1A and GSTP1 in HCC tissue of patients with HCV detected by pyrosequencing and methylation-specific PCR [90,110]. Multiple studies have found higher methylation ratios of p16 (79% versus 16%), APC (93% versus 20%) and RIZ1 (79% versus 9%) in HCV-related HCC as compared to paired non-tumour tissue [89,111]. Finally, SFRP2, a Wnt inhibitor, showed increased methylation levels in HCV-related HCC (n = 41) compared to cirrhosis, as detected by pyrosequencing on DNA from tissue [112].

Interestingly, HCV core protein influences the expression of SFRP1, RAR-β2 and E-cadherin through DNA methylation in HCC cell lines [113–115]. Also, higher levels of HCV-RNA have been associated with decreased methylation levels of MGMT [90], suggesting a direct correlation between viral activity and DNA methylation, which is exciting in terms of viral-related carcinogenesis but speaks of further detailing for biomarker specificity.

The use of cfDNA from plasma to monitor differences between HCV-related HCC and controls has also been conducted in a very limited fashion. Although it has been shown that higher cfDNA concentrations could be extracted from the plasma of HCV-positive HCC as compared to samples from cirrhosis and chronic HCV patients [116,117]. currently, only one study about specific DNA methylation on cfDNA in HCV-related HCC is available. The study found that in serum, the methylation ratio of p16 was higher in HCV-related HCC (n = 25, 92%) than in cirrhosis and chronic HCV samples [118], supporting the methylation data of p16 from HCC tissues [110].

### 5.4. DNA methylation markers in non-viral hepatitis-related HCC

Compared to viral hepatitis-related HCC, studies have found that DMMs in NAFLD- and ALD-related HCC are associated with alterations of metabolism-related pathways. Interestingly, chronic alcohol intake has been shown to impair retinoic acid homoeostasis [119], and studies have revealed retinol metabolism-related DMMs with decreased gene expression profiles in ALD-related HCC tissues [120]. This suggests that increased DNA methylation levels in response to alcohol use can regulate the expression of specific genes, and that these methylated markers may be involved in the development of ALD-related HCC. A study by Lambert et al. reported slightly higher methylation levels of MGMT in patients with viral hepatitis-related HCC with alcohol consumption as compared to a group without alcohol consumption (17% versus 11%) [90].

The available data on NAFLD-related HCC in one-carbon metabolism is mainly restricted to animal experiments and includes data on genes such as GNMT and MAT1A [121–123]. Only one study examined the methylation status of liver tissues obtained following partial hepatectomy, and compared NAFLD-related HCC (n = 22) with corresponding non-tumour tissues [91]. Six genes, including DCAF4L2, CKLF, UBE2C, TRIM4, PRC1 and TUBA1B, showed down-regulated methylation levels from normal liver tissues to paired non-tumourous tissues and to NAFLD-associated HCC tissue. Similarly, in mice fed with a high-fat diet, TUBB2B showed hypomethylation in the promoter region in correlation with an increased expression in NAFLD-related HCC [124]. However, more detailed studies for DMMs, especially in cfDNA, are needed in metabolic-related HCCs to grasp an understanding of the methylation-related mechanisms and potential biomarkers.

## 6. Final remarks

Over the last decade, numerous studies have been conducted to discover and validate novel non-invasive, blood biomarkers that can be used for screening high-risk patient populations in order to diagnose HCC at early stages. As described in this review, altered DNA methylation has been shown to progress in a dynamic fashion from healthy individuals to cirrhosis patients to patients with HCC [78]. Numerous studies have described the altered methylation status of genes in liver tissues of HCC patients as compared to surrounding non-HCC tissues, and mechanistic studies have demonstrated the importance of altered methylation of genes in the tumourigenesis process. However, as can be concluded from Fig. 3, which summarises the various studies described in this review, different distinctive DMMs are found depending on the characteristics of the tumour and the type of clinical material examined.

Much effort has been invested in examining the applicability of DMMs as novel biomarkers, especially for early-stage HCC using cfDNA isolated from blood. The findings of these studies are highly promising, and panels of different DMMs have been identified that perform equally well or better than other biomarkers, including AFP and the GALAD score, thereby opening the possibility to use liquid biopsy in early-stage HCC diagnosis. Some studies report AUC-values for these multi-marker panels in the range of 0.88–0.92 for early-stage HCC [76,77]. However, a number of important issues need to be resolved before DMMs can be broadly applied for HCC detection. One of the issues is related to the sensitivity of the assay, especially in early-stage HCC. At this stage, the tumour is small, and hence the contribution of ctDNA within cfDNA is quite limited. In addition, bisulfite conversion of DNA causes DNA degradation and loss, which, combined with the low starting point of ctDNA necessitates the use of high volumes of plasma for the assay in order to be above the lower limit of DMM detection. It has been reported that even with a blood draw of 10 mL, ctDNA was not always detected in very early-stage tumours [125]. Another issue that is highly relevant with respect to the identification of DMM as biomarkers is the heterogeneity of HCC as a result of genetic diversity, and as a result of the diversity of underlying liver diseases such as viral hepatitis, alcoholic and non-alcoholic liver disease. Moreover, the fact that HCC can arise in non-cirrhotic livers further complicates the use of a reliable and specific DMM panels. However, given the highly promising results that have been published to date and given the abundance of new technologies and methodologies that increase the sensitivity of the assays, it is highly likely that DMM detection in liquid biopsies will be one of the tools aiding in early-HCC detection in the years to come.

## Figures and Tables

**Fig. 1.**
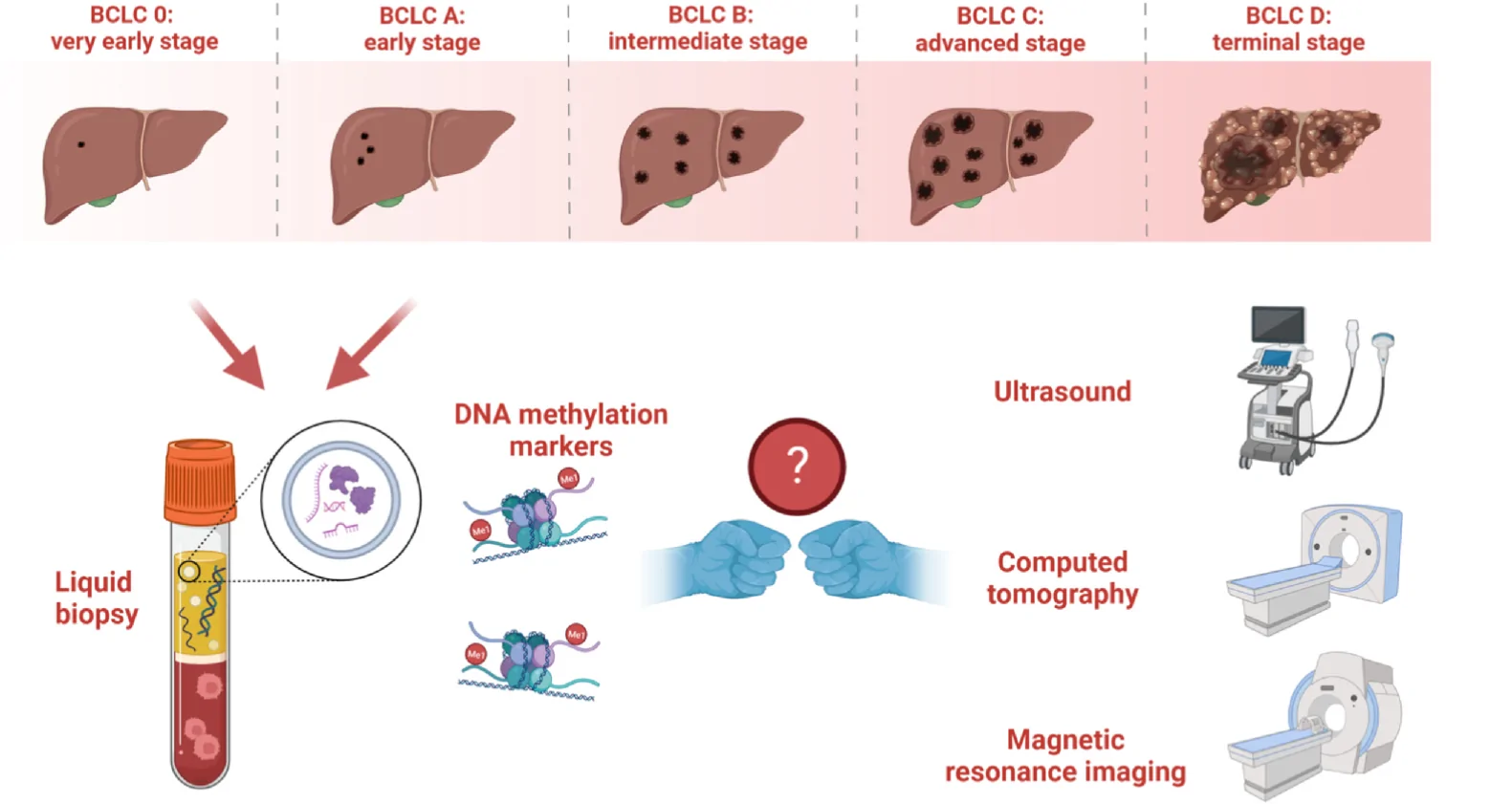
Techniques for HCC detection. Early-stage HCC refers to patients diagnosed with BCLC 0 or A stage. However, current techniques, including imaging do not perform well for the diagnosis of early-stage HCC. Liquid biopsy holds great promise since tumour-related products can be found and extracted from blood, such as DNA methylation markers (DMMs) using circulating tumour DNA. BCLC, Barcelona Clinic Liver Cancer; HCC, hepatocellular carcinoma.

**Fig. 2.**
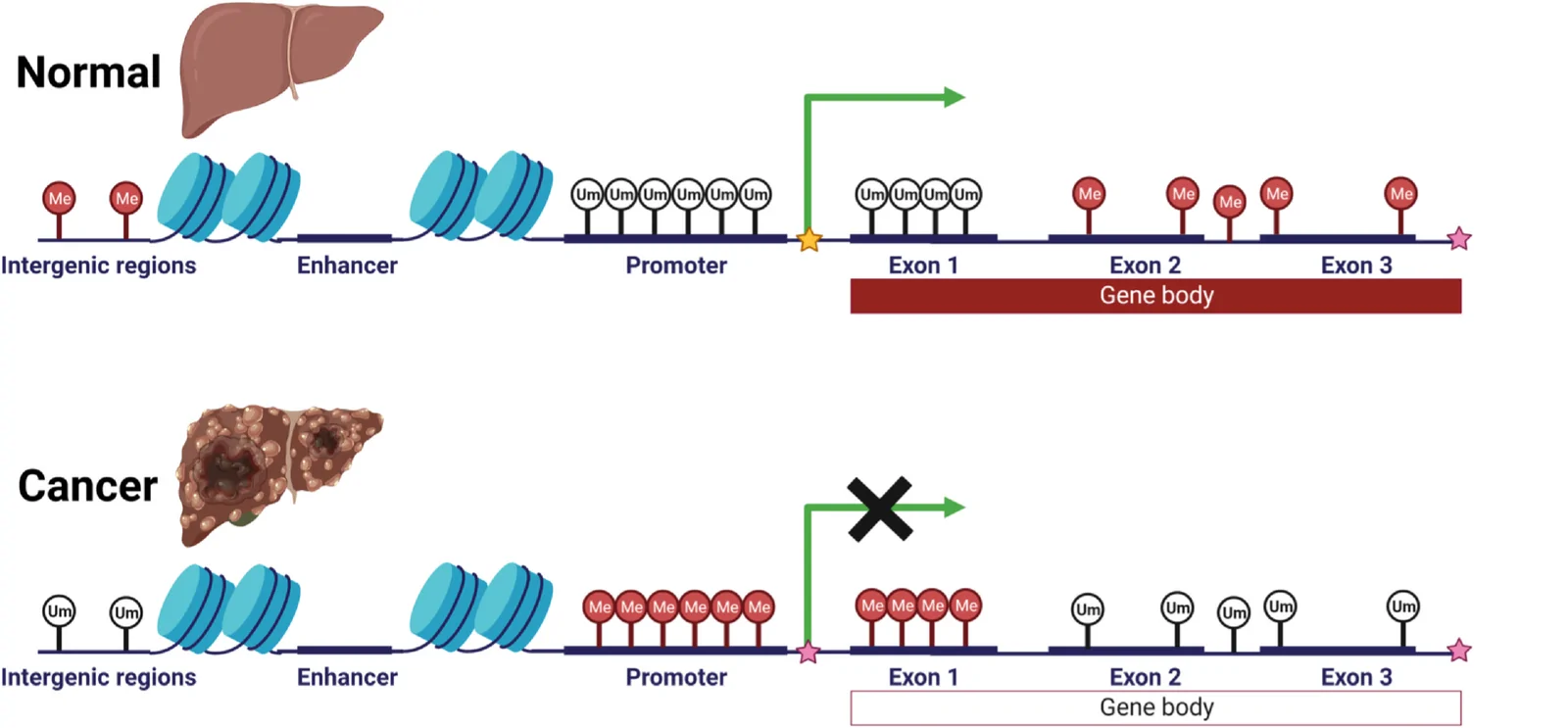
The differences in DNA methylation status in normal and cancerous HCC tissue. The CpG sites are regions of DNA with repetitive cytosines and guanines in a linear sequence of bases along 5′ to 3′ direction. In mammals, CpG sites are dispersed throughout the genome or accumulated to form CpG islands near the transcriptional regulatory regions. Under normal circumstances, the promoter and first exon regions are virtually unmethylated, while other regions, such as gene bodies, intergenic regions, and repeated elements, are methylated. In contrast, in cancer, relatively high methylation rates are observed in the promotor and first exon regions, and methylation of those regions will inhibit the interaction of transcription factors in these regions. CpGs, cytosine-guanine dinucleotides; HCC, hepatocellular carcinoma.

**Fig. 3.**
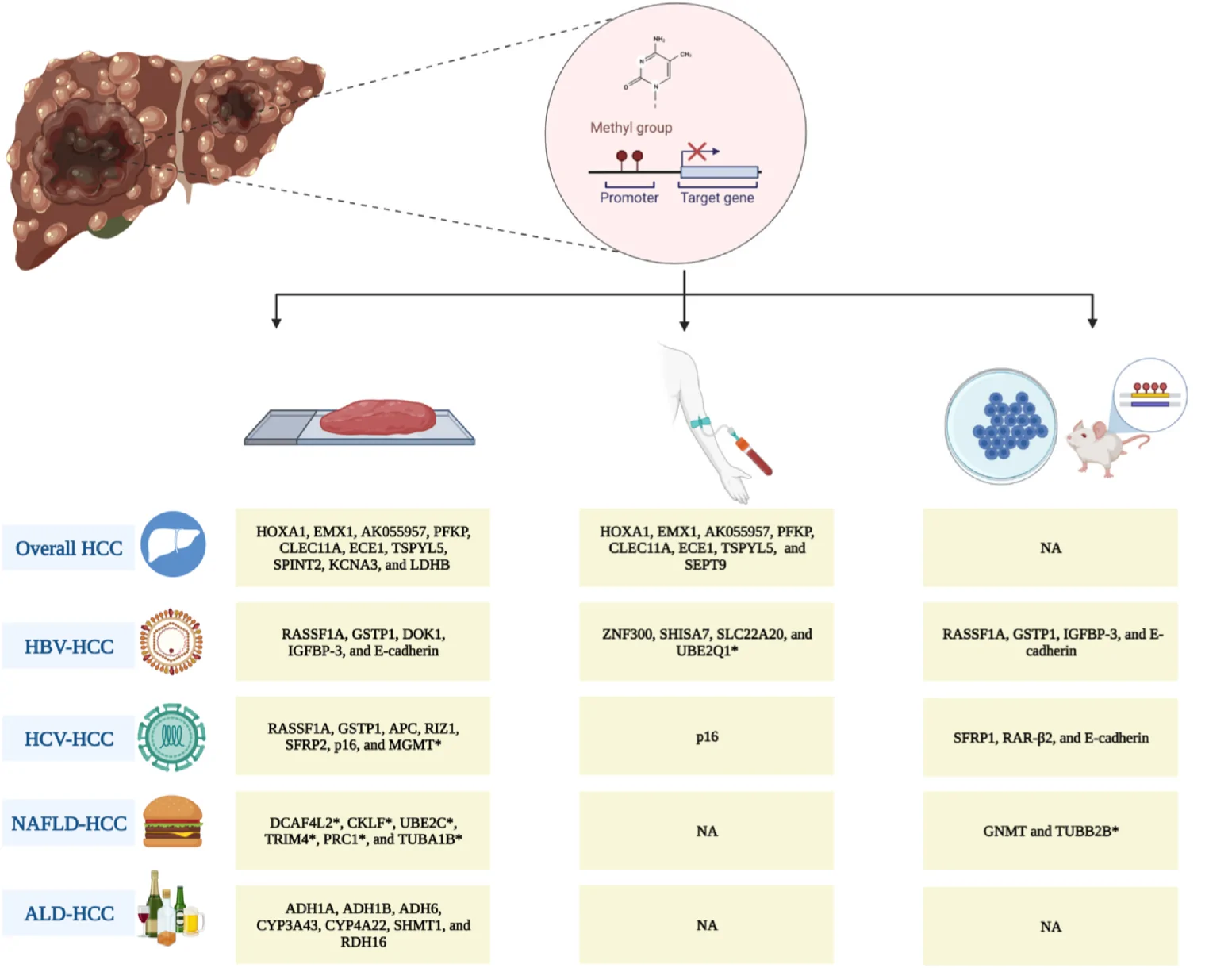
A summary of the DNA methylation markers described in this review that may differentiate HCC from non-HCC samples. The currently available data for DMMs in HCC: the biomarkers presented may not necessarily be specific to only one aetiology-related HCC since most of the studies did not perform genome-wide methylation profiling. In an overall heterogeneous HCC population, HOXA1, EMX1, AK055957, PFKP, CLEC11A, ECE1, TSPYL5, SPINT2, KCNA3, LDHB, and B3GALT6 are methylated in tissues; HOXA1, EMX1, AK055957, PFKP, CLEC11A, ECE1, TSPYL5, SEPT9, and B3GALT6 are methylated in blood. In HBV-related HCC, RASSF1A, GSTP1, DOK1, IGFBP-3, and E-cadherin are methylated in tissues; ZNF300, UBE2Q1, SHISA7, and SLC22A20 are methylated in blood; RASSF1A, GSTP1, IGFBP-3, and E-cadherin are methylated in HCC cell lines. In HCV-related HCC, RASSF1A, GSTP1, APC, RIZ1, SFRP2, MGMT, and p16 are methylated in tissues; p16 is methylated in blood; SFRP1, RAR-β2, and E-cadherin are methylated in HCC cell lines. In NAFLD-related HCC, DCAF4L2, CKLF, UBE2C, TRIM4, PRC1, and TUBA1B are methylated in tissues; GNMT and TUBB2B are methylated in mice livers. In ALD-related HCC, ADH1A, ADH1B, ADH6, CYP3A43, CYP4A22, SHMT1, and RDH16 are methylated in tissues. APC, adenomatous polyposis coli; ADH1A, alcohol dehydrogenase type 1A gene; ADH1B, alcohol dehydrogenase 2; ADH6, alcohol dehydrogenase 6; ALD, alcoholic liver disease; CYP3A43, cytochrome P450 3A43; CYP4A22, cytochrome P450 4A22; CKLF, chemokine-like factor; DOK1, downstream of tyrosine kinases 1; DCAF4L2, DDB1-and Cul4-associated factor 4-like 2; DMMs, DNA methylation markers; GNMT, glycine N-methyltransferase; GSTP1, glutathione S-transferase pi 1; HCC, hepatocellular carcinoma; HBV, hepatitis B virus; HCV, hepatitis C virus; IGFBP-3, insulin-like growth factor binding protein-3; MGMT, O6-methylguanine-methyltransferase; NA, not available; NAFLD, non-alcoholic fatty liver disease; p16, cyclin-Dependent Kinase Inhibitor p16; PRC1, Polycomb repressive complex 1; RASSF1A, Ras associated domain family 1A; RAR-β2, retinoic acid receptor-beta2; RIZ1, retinoblastoma protein-interacting zinc-finger gene 1; RDH16, retinol dehydrogenase 16; SHISA7, shisa family member 7; SLC22A20, organic anion transporter; SFRP1, secreted frizzled-related protein 1; SFRP2, secreted frizzled-related protein 2; SHMT1, serine hydroxymethyltransferase 1; TRIM4, tripartite motif-containing 4; TUBA1B, tubulin alpha 1b; Tubb2b, tubulin, beta-2b; UBE2Q1, Ubiquitin-Conjugating Enzyme2 Q1; UBE2C, Ubiquitin conjugating enzyme E2C; ZNF300, zinc finger protein 300.*, hypomethylated.

**Table 1:** Current techniques for DNA methylation.

Techniques	DNA input (Available data)	CpG sites coverage	cfDNA available	Pros	Cons	Reference

Whole genome bisulfite sequencing	• gDNA: 1–5 μg • FFPE DNA: 1–5 μg	95%	No	• High CpGs coverage	• Expensive • High DNA input	[42,44]
Reduced representative bisulfite sequencing	• gDNA: 0.2–1 μg • FFPE DNA: 0.5–1 μg	14.2%	No	• Targeted on high CpGs density regions	• Expensive • Low CpGs coverage	[42,45,46]
Cell-free reduced representation bisulfite sequencing	• cfDNA: 0.75–10 ng	10.6%	Yes	• Low threshold for DNA input	• Low CpGs coverage	[47,48]
Pyrosequencing	• gDNA: 0.25–1 μg	NA	Yes	• Available for both CpGs-rich and CpGs-poor regions	• Only short regions can be measured	[49,50]
Illumina HM450	• gDNA: 0.5–1 μg • serum DNA: 0.5 μg	1.7%	Yes	• Easy to use (pre-designed panel)	• Low CpGs coverage	[42,44,51,53]
Methylated DNA sequencing	• cfDNA: 10 ng	58.3%	Yes	• High CpGs coverage • Without harsh bisulfite conversion	NA	[57,58]
TET-assisted pyridine borane sequencing	• gDNA: 1 ng • cfDNA: 1 ng	Similar to WGBS	Yes	• High CpGs coverage • Without harsh bisulfite conversion	NA	[59,60]
Methyl-DNA immunoprecipitation sequencing	• gDNA: 50 ng • cfDNA: 1–10 ng	87.7%	Yes	• High CpGs coverage • Without harsh bisulfite conversion	• Low resolution (∼150 bp)	[61–64]
Methyl-binding domain capture sequencing	• gDNA: 0.2–1 μg • FFPE DNA: 0.5–1 μg • cfDNA: 30 ng	17.7%	Yes	• Do not interact with 5hmC. • Without harsh bisulfite conversion	• Low resolution • Low CpGs coverage	[42,106]
Quantitative methylation-specific PCR	• cfDNA: 5 ng	NA	Yes	• Easy to apply in clinical practice • Cost-effective	• Harsh assay design and standardisations	[50,57]

cfDNA, cell-free DNA; cf-RRBS, cell-free reduced representation bisulfite sequencing; CpGs, cytosine-guanine dinucleotides; FFPE DNA, formalin-fixed paraffin-embedded DNA; genomic DNA, gDNA; Illumina HM450, Illumina Infinium Human Methylation 450 Bead Chip array; MeD-seq, methylated DNA sequencing; MeDIP-Seq, methyl-DNA immunoprecipitation sequencing; MBDCap-Seq, methyl-binding domain capture sequencing; qMSP, quantitative methylation-specific PCR; RRBS, reduced representative bisulfite sequencing; TAPS, TET-assisted pyridine borane sequencing; WGBS, whole genome bisulfite sequencing.

**Table 2:** Selection of relatively large studies of DMMs in early-stage HCC.

DMMs	Sources (Volume)	Testing samples (n)	Comments	Reference

10 DMMs	Plasma (1.5 mL)	• 1098 HCC • 835 normal individuals	• This model showed high AUC (> 0.94) for HCC detection compared to normal controls, which is better than AFP (0.82).	[70]
HOXA1, EMX1, AK055957, PFKP, CLEC11A, ECE1, and B3GALT6	Plasma (> 1 mL)	• 95 HCC (48% early-stage) • 51 cirrhosis without HCC • 98 controls without cirrhosis	• This panel detected 75% (n = 4) BCLC-0, and 93% (n = 42) BCLC-A HCC.	[20]
HOXA1, EMX1, TSPYL5, and B3GALT6	Plasma (3 mL)	• 135 HCC (56% early-stage) • 302 controls (87% cirrhosis)	• In combination with AFP and AFP-L3, this panel achieved 71% early-stage sensitivity for HCC, which is much higher than AFP, DCP and GALAD score.	[20,76]
HOXA1, TSPYL5, and B3GALT6	Plasma (5–6 mL)	• 156 HCC (50% early-stage) • 245 controls (92% cirrhosis)	• In combination with AFP and gender, this panel exhibited 82% sensitivity for early-stage HCC, better than AFP and GALAD score.	[20,77]
TSPYL5, SPINT2, KCNA3, and LDHB	Liver tissues	• 227 HCC (87% early-stage) • 8 dysplastic nodules • 139 cirrhosis • 16 healthy controls	• These four DMMs may be epigenetic gatekeeper candidates because of the increased proportion trend in the development of HCC with confirmed inverse correlation between DNA methylation and gene expression.	[78]
SEPT9	Plasma (3.5 mL)	• 98 HCC with cirrhosis (31% early-stage) • 191 cirrhosis without HCC	• To diagnose BCLC A HCC, the mSEPT9 test exhibited 64–88% sensitivity and 68–95% specificity.	[84,85]
SEPT9	Serum (0.4 mL)	• 136 HCC (45% early-stage) • 45 chronic liver disease • 80 healthy individuals	• The median copy numbers of methylated SEPT9 showed a dynamic trend from 0 in healthy control, 2.0 in chronic liver disease groups, to 6.4 in HCC groups.	[86]
HelioLiver Test (28 DMMs)	Plasma (NA)	• 122 HCC (37% early-stage) • 125 controls (37% cirrhosis)	• The algorithm exhibited 76% sensitivity for early-stage (stage I and II) HCC, better than AFP and GALAD score.	[81]
5-hmC diagnostic model	Plasma (5–10 mL)	• 809 HCC (27.1% early-stage) • 129 cirrhosis and chronic hepatitis B • 256 controls	• The model showed high AUC for early-stage HCC both in the training cohort (87.3%) and the validation cohort (84.6%); better than AFP (79.3% and 69.2%, respectively).	[82]

AFP, alfa-fetoprotein; BCLC, Barcelona clinic liver cancer; B3GALT6, beta-1,3-galactosyltransferase 6; CLEC11A, C-type lectin domain containing 11A; DCP, des-carboxy-prothrombin; DMMs, DNA methylation markers; EMX1, empty spiracles homeobox 1; ECE1, endothelin-converting enzyme 1; HCC, hepatocellular carcinoma; HOXA1, homeobox A1; KCNA3, potassium voltage-gated channel, shaker-related subfamily, member 3; LDHB, lactate dehydrogenase B; mSEPT9, methylated SEPT9; NA, not available; PFKP, phosphofructokinase; SPINT2, serine peptidase inhibitor, Kunitz type 2; SEPT9, septin 9 gene; TSPYL5, testis-specific Y-encoded-like protein 5; 5-hmC, 5-hydroxymethylcytosine.

## References

[1] Akinyemiju T, Abera S, Ahmed M, Alam N, Alemayohu MA, Allen C, The burden of primary liver cancer and underlying etiologies from 1990 to 2015 at the global, regional, and national level: results from the global burden of disease study 2015. JAMA Oncol 2017;3(12):1683–91. 10.1001/jamaoncol.2017.3055 PMC5824275 28983565

[2] Desai A, Sandhu S, Lai JP, Sandhu DS. Hepatocellular carcinoma in non-cirrhotic liver: a comprehensive review. World J Hepatol 2019;11(1):1–18. 10.4254/wjh.v11.i1.1 PMC6354117 30705715

[3] Papatheodoridis GV, Idilman R, Dalekos GN, Buti M, Chi H, van Boemmel F, The risk of hepatocellular carcinoma decreases after the first 5 years of entecavir or tenofovir in Caucasians with chronic hepatitis B. Hepatology 2017;66(5):1444–53. 10.1002/hep.29320 28622419

[4] Kanwal F, Kramer J, Asch SM, Chayanupatkul M, Cao Y, El-Serag HB. Risk of hepatocellular cancer in HCV patients treated with direct-acting antiviral agents. Gastroenterology 2017;153(4):996–1005. e1001. 10.1053/j.gastro.2017.06.012 28642197

[5] McGlynn KA, Petrick JL, London WT. Global epidemiology of hepatocellular carcinoma: an emphasis on demographic and regional variability. Clin Liver Dis 2015;19(2):223–38. 10.1016/j.cld.2015.01.001 PMC4712629 25921660

[6] Humans IWGotEoCRt. Biological agents. Volume 100 B. A review of human carcinogens. IARC Monogr Eval Carcinog Risks Hum 2012;100(Pt B):1–441. PMC4781184 23189750

[7] De Mitri MS, Poussin K, Baccarini P, Pontisso P, D’Errico A, Simon N, HCV-associated liver cancer without cirrhosis. Lancet 1995;345(8947):413–5. 10.1016/s0140-6736(95)90400-x 7772123

[8] Estes C, Razavi H, Loomba R, Younossi Z, Sanyal AJ. Modeling the epidemic of non-alcoholic fatty liver disease demonstrates an exponential increase in burden of disease. Hepatology 2018;67(1):123–33. 10.1002/hep.29466 PMC5767767 28802062

[9] Llovet JM, Kelley RK, Villanueva A, Singal AG, Pikarsky E, Roayaie S, Hepatocellular carcinoma. Nat Rev Dis Primers 2021;7(1):6. 10.1038/s41572-020-00240-3 PMC13537433 33479224

[10] Kulik L, El-Serag HB. Epidemiology and management of hepatocellular carcinoma. Gastroenterology 2019;156(2):477–91. e471. 10.1053/j.gastro.2018.08.065 PMC6340716 30367835

[11] Reig M, Forner A, Rimola J, Ferrer-Fabrega J, Burrel M, Garcia-Criado A, BCLC strategy for prognosis prediction and treatment recommendation: the 2022 update. J Hepatol 2022;76(3):681–93. 10.1016/j.jhep.2021.11.018 PMC8866082 34801630

[12] Llovet JM, Zucman-Rossi J, Pikarsky E, Sangro B, Schwartz M, Sherman M, Hepatocellular carcinoma. Nat Rev Dis Primers 2016;2:16018. 10.1038/nrdp.2016.18 27158749

[13] Villanueva A Hepatocellular carcinoma. N Engl J Med 2019;380(15):1450–62. 10.1056/NEJMra1713263 30970190

[14] Gupta S, Bent S, Kohlwes J. Test characteristics of alpha-fetoprotein for detecting hepatocellular carcinoma in patients with hepatitis C. A systematic review and critical analysis. Ann Intern Med 2003;139(1):46–50. 10.7326/0003-4819-139-1-200307010-00012 12834318

[15] Tzartzeva K, Obi J, Rich NE, Parikh ND, Marrero JA, Yopp A, Surveillance imaging and alpha fetoprotein for early detection of hepatocellular carcinoma in patients with cirrhosis: a meta-analysis. Gastroenterology 2018;154(6):1706–18. e1701. 10.1053/j.gastro.2018.01.064 PMC5927818 29425931

[16] Beudeker BJB, Boonstra A. Circulating biomarkers for early detection of hepatocellular carcinoma. Therap Adv Gastroenterol 2020;13. 1756284820931734. 10.1177/1756284820931734 PMC7325534 32647536

[17] Yang JD, Addissie BD, Mara KC, Harmsen WS, Dai J, Zhang N, GALAD score for hepatocellular carcinoma detection in comparison with liver ultrasound and proposal of GALADUS score. Cancer Epidemiol Biomarkers Prev 2019;28(3):531–8. 10.1158/1055-9965.EPI-18-0281 PMC6401221 30464023

[18] Marzese DM, Hirose H, Hoon DS. Diagnostic and prognostic value of circulating tumour-related DNA in cancer patients. Expert Rev Mol Diagn 2013;13(8):827–44. 10.1586/14737159.2013.845088 24127721

[19] Tischoff I, Tannapfe A. DNA methylation in hepatocellular carcinoma. World J Gastroenterol 2008;14(11):1741–8. 10.3748/wjg.14.1741 PMC2695914 18350605

[20] Kisiel JB, Dukek BA, VSRK R, Ghoz HM, Yab TC, Berger CK, Hepatocellular carcinoma detection by plasma methylated DNA: discovery, phase I pilot, and phase II clinical validation. Hepatology 2019;69(3):1180–92. 10.1002/hep.30244 PMC6429916 30168613

[21] Catania S, Dumesic PA, Pimentel H, Nasif A, Stoddard CI, Burke JE, Evolutionary persistence of DNA methylation for millions of years after ancient loss of a *de novo* methyltransferase. Cell 2020;180(2):263–77. e220. 10.1016/j.cell.2019.12.012 PMC7197499 31955845

[22] Law JA, Jacobsen SE. Establishing, maintaining and modifying DNA methylation patterns in plants and animals. Nat Rev Genet 2010;11(3):204–20. 10.1038/nrg2719 PMC3034103 20142834

[23] Kulis M, Esteller M. DNA methylation and cancer. Adv Genet 2010;70:27–56. 10.1016/B978-0-12-380866-0.60002-2 20920744

[24] Gardiner-Garden M, Frommer M. CpG islands in vertebrate genomes. J Mol Biol 1987;196(2):261–82. 10.1016/0022-2836(87)90689-9 3656447

[25] Chen QW, Zhu XY, Li YY, Meng ZQ. Epigenetic regulation and cancer (review). Oncol Rep 2014;31(2):523–32. 10.3892/or.2013.2913 24337819

[26] Bogdanovic O, Veenstra GJ. DNA methylation and methyl-CpG binding proteins: developmental requirements and function. Chromosoma 2009;118(5):549–65. 10.1007/s00412-009-0221-9 PMC2729420 19506892

[27] Klose RJ, Bird AP. Genomic DNA methylation: the mark and its mediators. Trends Biochem Sci 2006;31(2):89–97. 10.1016/j.tibs.2005.12.008 16403636

[28] Singal R, Wang SZ, Sargent T, Zhu SZ, Ginder GD. Methylation of promoter proximal-transcribed sequences of an embryonic globin gene inhibits transcription in primary erythroid cells and promotes formation of a cell type-specific methyl cytosine binding complex. J Biol Chem 2002;277(3):1897–905. 10.1074/jbc.M105580200 11684679

[29] Takasawa K, Arai Y, Yamazaki-Inoue M, Toyoda M, Akutsu H, Umezawa A, DNA hypermethylation enhanced telomerase reverse transcriptase expression in human-induced pluripotent stem cells. Hum Cell 2018;31(1):78–86. 10.1007/s13577-017-0190-x 29103143

[30] Wu X, Zhang Y. TET-mediated active DNA demethylation: mechanism, function and beyond. Nat Rev Genet 2017;18(9):517–34. 10.1038/nrg.2017.33 28555658

[31] Esteller M Epigenetics in cancer. N Engl J Med 2008;358(11):1148–59. 10.1056/NEJMra072067 18337604

[32] Rodriguez-Martin B, Alvarez EG, Baez-Ortega A, Zamora J, Supek F, Demeulemeester J, Pan-cancer analysis of whole genomes identifies driver rearrangements promoted by LINE-1 retrotransposition. Nat Genet 2020;52(3):306–19. 10.1038/s41588-019-0562-0 PMC7058536 32024998

[33] Schauer SN, Carreira PE, Shukla R, Gerhardt DJ, Gerdes P, Sanchez-Luque FJ, L1 retrotransposition is a common feature of mammalian hepatocarcinogenesis. Genome Res 2018;28(5):639–53. 10.1101/gr.226993.117 PMC5932605 29643204

[34] Shukla R, Upton KR, Munoz-Lopez M, Gerhardt DJ, Fisher ME, Nguyen T, Endogenous retrotransposition activates oncogenic pathways in hepatocellular carcinoma. Cell 2013;153(1):101–11. 10.1016/j.cell.2013.02.032 PMC3898742 23540693

[35] Xia J, Han L, Zhao Z. Investigating the relationship of DNA methylation with mutation rate and allele frequency in the human genome. BMC Genomics 2012;13(Suppl 8):S7. 10.1186/1471-2164-13-S8-S7 PMC3535710 23281708

[36] Mugal CF, Ellegren H. Substitution rate variation at human CpG sites correlates with non-CpG divergence, methylation level and GC content. Genome Biol 2011;12(6):R58. 10.1186/gb-2011-12-6-r58 PMC3218846 21696599

[37] Mahon KL, Qu W, Devaney J, Paul C, Castillo L, Wykes RJ, Methylated Glutathione S-transferase 1 (mGSTP1) is a potential plasma free DNA epigenetic marker of prognosis and response to chemotherapy in castrate-resistant prostate cancer. Br J Cancer 2014;111(9):1802–9. 10.1038/bjc.2014.463 PMC4453725 25144624

[38] Costa FF, Paixao VA, Cavalher FP, Ribeiro KB, Cunha IW, Rinck JA Jr., SATR-1 hypomethylation is a common and early event in breast cancer. Cancer Genet Cytogenet 2006;165(2):135–43. 10.1016/j.cancergencyto.2005.07.023 16527607

[39] Gouil Q, Keniry A. Latest techniques to study DNA methylation. Essays Biochem 2019;63(6):639–48. 10.1042/EBC20190027 PMC6923321 31755932

[40] Darst RP, Pardo CE, Ai L, Brown KD, Kladde MP. Bisulfite sequencing of DNA. Curr Protoc Mol Biol 2010(9):1–17 Chapter 7:Unit 7. 10.1002/0471142727.mb0709s91 PMC3214597 20583099

[41] Galardi F, Luca F, Romagnoli D, Biagioni C, Moretti E, Biganzoli L, Cell-free DNA-methylation-based methods and applications in oncology. Biomolecules 2020;10:12. 10.3390/biom10121677 PMC7765488 33334040

[42] Stirzaker C, Taberlay PC, Statham AL, Clark SJ. Mining cancer methylomes: prospects and challenges. Trends Genet 2014;30(2):75–84. 10.1016/j.tig.2013.11.004 24368016

[43] Zhou L, Ng HK, Drautz-Moses DI, Schuster SC, Beck S, Kim C, Systematic evaluation of library preparation methods and sequencing platforms for high-throughput whole genome bisulfite sequencing. Sci Rep 2019;9(1):10383. 10.1038/s41598-019-46875-5 PMC6637168 31316107

[44] Zhou L, Ng HK, Drautz-Moses DI, Schuster SC, Beck S, Kim C, Systematic evaluation of library preparation methods and sequencing platforms for high-throughput whole genome bisulfite sequencing. Sci Rep 2019;9(1):10383. 10.1038/s41598-019-46875-5 PMC6637168 31316107

[45] Gu H, Bock C, Mikkelsen TS, Jager N, Smith ZD, Tomazou E, Genome-scale DNA methylation mapping of clinical samples at single-nucleotide resolution. Nat Methods 2010;7(2):133–6. 10.1038/nmeth.1414 PMC2860480 20062050

[46] Veillard A-C, Datlinger P, Laczik M, Squazzo S, Bock C. Diagenode premium RRBS technology: cost-effective DNA methylation mapping with superior coverage. Nat Methods 2016;13(2):i–.

[47] Van Paemel R, De Koker A, Vandeputte C, van Zogchel L, Lammens T, Laureys G, Minimally invasive classification of paediatric solid tumours using reduced representation bisulphite sequencing of cell-free DNA: a proof-of-principle study. Epigenetics 2021;16(2):196–208. 10.1080/15592294.2020.1790950 PMC7889189 32662719

[48] De Koker A, Van Paemel R, De Wilde B, De Preter K, Callewaert N. A versatile method for circulating cell-free DNA methylome profiling by reduced representation bisulfite sequencing. bioRxiv 2019:663195. 10.1101/663195.

[49] Delaney C, Garg SK, Yung R. Analysis of DNA methylation by pyrosequencing. Methods Mol Biol 2015;1343:249–64. 10.1007/978-1-4939-2963-4_19 PMC4772880 26420722

[50] Sestakova S, Salek C, Remesova H. DNA methylation validation methods: a coherent review with practical comparison. Biol Proced Online 2019;21:19. 10.1186/s12575-019-0107-z PMC6771119 31582911

[51] Huang YW, Huang TH, Wang LS. Profiling DNA methylomes from microarray to genome-scale sequencing. Technol Cancer Res Treat 2010;9(2):139–47. 10.1177/153303461000900203 PMC3011833 20218736

[52] Kurdyukov S, Bullock M. DNA methylation analysis: choosing the right method. Biology (Basel) 2016;5:1. 10.3390/biology5010003 PMC4810160 26751487

[53] Zhang P, Wen X, Gu F, Deng X, Li J, Dong J, Methylation profiling of serum DNA from hepatocellular carcinoma patients using an Infinium Human Methylation 450 BeadChip. Hepatol Int 2013;7(3):893–900. 10.1007/s12072-013-9437-0 26201927

[54] Hoadley KA, Yau C, Hinoue T, Wolf DM, Lazar AJ, Drill E, Cell-of-origin patterns dominate the molecular classification of 10,000 tumours from 33 types of cancer. Cell 2018;173(2):291–304. e296. 10.1016/j.cell.2018.03.022 PMC5957518 29625048

[55] Wu Z, Bai Y, Cheng Z, Liu F, Wang P, Yang D, Absolute quantification of DNA methylation using microfluidic chip-based digital PCR. Biosens Bioelectron 2017;96:339–44. 10.1016/j.bios.2017.05.021 28525852

[56] Ball MP, Li JB, Gao Y, Lee JH, LeProust EM, Park IH, Targeted and genome-scale strategies reveal gene-body methylation signatures in human cells. Nat Biotechnol 2009;27(4):361–8. 10.1038/nbt.1533 PMC3566772 19329998

[57] Deger T, Boers RG, de Weerd V, Angus L, van der Put MMJ, Boers JB, High-throughput and affordable genome-wide methylation profiling of circulating cell-free DNA by methylated DNA sequencing (MeD-seq) of LpnPI digested fragments. Clin Epigenetics 2021;13(1):196. 10.1186/s13148-021-01177-4 PMC8529776 34670587

[58] Boers R, Boers J, de Hoon B, Kockx C, Ozgur Z, Molijn A, Genome-wide DNA methylation profiling using the methylation-dependent restriction enzyme LpnPI. Genome Res 2018;28(1):88–99. 10.1101/gr.222885.117 PMC5749185 29222086

[59] Liu Y, Siejka-Zielinska P, Velikova G, Bi Y, Yuan F, Tomkova M, Bisulfite-free direct detection of 5-methylcytosine and 5-hydroxymethylcytosine at base resolution. Nat Biotechnol 2019;37(4):424–9. 10.1038/s41587-019-0041-2 30804537

[60] Siejka-Zielinska P, Cheng J, Jackson F, Liu Y, Soonawalla Z, Reddy S, Cell-free DNA TAPS provides multimodal information for early cancer detection. Sci Adv 2021;7(36):eabh0534. 10.1126/sciadv.abh0534 PMC8442905 34516908

[61] Hsu HK, Weng YI, Hsu PY, Huang TH, Huang YW. Detection of DNA methylation by MeDIP and MBDCap assays: an overview of techniques. Methods Mol Biol 2020;2102:225–34. 10.1007/978-1-0716-0223-2_12 31989558

[62] Clark C, Palta P, Joyce CJ, Scott C, Grundberg E, Deloukas P, A comparison of the whole genome approach of MeDIP-seq to the targeted approach of the Infinium HumanMethylation450 BeadChip(R) for methylome profiling. PLoS One 2012;7(11). e50233. 10.1371/journal.pone.0050233 PMC3510246 23209683

[63] Shen SY, Burgener JM, Bratman SV, De, Carvalho DD. Preparation of cfMeDIP-seq libraries for methylome profiling of plasma cell-free DNA. Nat Protoc 2019;14(10):2749–80. 10.1038/s41596-019-0202-2 31471598

[64] Xing X, Zhang B, Li D, Wang T. Comprehensive whole DNA Methylome Analysis by Integrating MeDIP-seq and MRE-seq. Methods Mol Biol 2018;1708:209–46. 10.1007/978-1-4939-7481-8_12 PMC6007844 29224147

[65] Guo S, Diep D, Plongthongkum N, Fung HL, Zhang K, Zhang K. Identification of methylation haplotype blocks aids in deconvolution of heterogeneous tissue samples and tumour tissue-of-origin mapping from plasma DNA. Nat Genet 2017;49(4):635–42. 10.1038/ng.3805 PMC5374016 28263317

[66] Moss J, Magenheim J, Neiman D, Zemmour H, Loyfer N, Korach A, Comprehensive human cell-type methylation atlas reveals origins of circulating cell-free DNA in health and disease. Nat Commun 2018;9(1):5068. 10.1038/s41467-018-07466-6 PMC6265251 30498206

[67] Diehl F, Schmidt K, Choti MA, Romans K, Goodman S, Li M, Circulating mutant DNA to assess tumour dynamics. Nat Med 2008;14(9):985–90. 10.1038/nm.1789 PMC2820391 18670422

[68] Pittella-Silva F, Chin YM, Chan HT, Nagayama S, Miyauchi E, Low SK, Plasma or serum: which is preferable for mutation detection in liquid biopsy? Clin Chem 2020;66(7):946–57. 10.1093/clinchem/hvaa103 32516802

[69] Wan JCM, Massie C, Garcia-Corbacho J, Mouliere F, Brenton JD, Caldas C, Liquid biopsies come of age: towards implementation of circulating tumour DNA. Nat Rev Cancer 2017;17(4):223–38. 10.1038/nrc.2017.7 28233803

[70] Xu RH, Wei W, Krawczyk M, Wang W, Luo H, Flagg K, Circulating tumour DNA methylation markers for diagnosis and prognosis of hepatocellular carcinoma. Nat Mater 2017;16(11):1155–61. 10.1038/nmat4997 29035356

[71] Luo B, Ma F, Liu H, Hu J, Rao L, Liu C, Cell-free DNA methylation markers for differential diagnosis of hepatocellular carcinoma. BMC Med 2022;20(1):8. 10.1186/s12916-021-02201-3 PMC8759185 35027051

[72] Wen L, Li J, Guo H, Liu X, Zheng S, Zhang D, Genome-scale detection of hypermethylated CpG islands in circulating cell-free DNA of hepatocellular carcinoma patients. Cell Res 2015;25(11):1250–64. 10.1038/cr.2015.126 PMC4650428 26516143

[73] Liang W, Zhao Y, Huang W, Gao Y, Xu W, Tao J, Non-invasive diagnosis of early-stage lung cancer using high-throughput targeted DNA methylation sequencing of circulating tumour DNA (ctDNA). Theranostics 2019;9(7):2056–70. 10.7150/thno.28119 PMC6485294 31037156

[74] Lin WH, Xiao J, Ye ZY, Wei DL, Zhai XH, Xu RH, Circulating tumour DNA methylation marker MYO1-G for diagnosis and monitoring of colorectal cancer. Clin Epigenetics 2021;13(1):232. 10.1186/s13148-021-01216-0 PMC8713401 34961566

[75] Beudeker BJB, Groothuismink ZMA, de Man RA, Witjes CDM, van der Eijk AA, Boonstra A, Hepatitis B core-related antigen levels predict recurrence-free survival in patients with HBV-associated early-stage hepatocellular carcinoma: results from a Dutch long-term follow-up study. J Viral Hepat 2021;28(1):205–8. 10.1111/jvh.13394 PMC7756674 32869922

[76] Chalasani NP, Ramasubramanian TS, Bhattacharya A, Olson MC, Edwards VD, Roberts LR, A Novel blood-based panel of methylated DNA and protein markers for detection of early-stage hepatocellular carcinoma. Clin Gastroenterol Hepatol 2021;19(12):2597–605. e2594. 10.1016/j.cgh.2020.08.065 32889146

[77] Chalasani NP, Porter K, Bhattacharya A, Book AJ, Neis BM, Xiong KM, Validation of a novel multitarget blood test shows high sensitivity to detect early stage hepatocellular carcinoma. Clin Gastroenterol Hepatol 2022;20(1):173–82. e177. 10.1016/j.cgh.2021.08.010 34391922

[78] Hernandez-Meza G, von Felden J, Gonzalez-Kozlova EE, Garcia-Lezana T, Peix J, Portela A, DNA methylation profiling of human hepatocarcinogenesis. Hepatology 2021;74(1):183–99. 10.1002/hep.31659 PMC8144238 33237575

[79] Qiu X, Hu B, Huang Y, Deng Y, Wang X, Zheng F. Hypermethylation of ACP1, BMP4, and TSPYL5 in hepatocellular carcinoma and their potential clinical significance. Dig Dis Sci 2016;61(1):149–57. 10.1007/s10620-015-3878-3 26386860

[80] Shen J, Wang S, Zhang YJ, Kappil M, Wu HC, Kibriya MG, Genome-wide DNA methylation profiles in hepatocellular carcinoma. Hepatology 2012;55(6):1799–808. 10.1002/hep.25569 PMC3330167 22234943

[81] Lin N, Lin Y, Xu J, Liu D, Li D, Meng H, A multi-analyte cell-free DNA-based blood test for early detection of hepatocellular carcinoma. Hepatol Commun 2022;6(7):1753–63. 10.1002/hep4.1918 PMC9234637 35244350

[82] Cai J, Chen L, Zhang Z, Zhang X, Lu X, Liu W, Genome-wide mapping of 5-hydroxymethylcytosines in circulating cell-free DNA as a non-invasive approach for early detection of hepatocellular carcinoma. Gut 2019;68(12):2195–205. 10.1136/gutjnl-2019-318882 PMC6872444 31358576

[83] Potter NT, Hurban P, White MN, Whitlock KD, Lofton-Day CE, Tetzner R, Validation of a real-time PCR-based qualitative assay for the detection of methylated SEPT9 DNA in human plasma. Clin Chem 2014;60(9):1183–91. 10.1373/clinchem.2013.221044 24938752

[84] Villanueva A, Portela A, Sayols S, Battiston C, Hoshida Y, Mendez-Gonzalez J, DNA methylation-based prognosis and epidrivers in hepatocellular carcinoma. Hepatology 2015;61(6):1945–56. 10.1002/hep.27732 PMC12337117 25645722

[85] Oussalah A, Rischer S, Bensenane M, Conroy G, Filhine-Tresarrieu P, Debard R, Plasma mSEPT9: a novel circulating cell-free DNA-based epigenetic biomarker to diagnose hepatocellular carcinoma. EBioMedicine 2018;30:138–47. 10.1016/j.ebiom.2018.03.029 PMC5952996 29627389

[86] Kotoh Y, Suehiro Y, Saeki I, Hoshida T, Maeda M, Iwamoto T, Novel liquid biopsy test based on a sensitive methylated SEPT9 assay for diagnosing hepatocellular carcinoma. Hepatol Commun 2020;4(3):461–70. 10.1002/hep4.1469 PMC7049671 32140662

[87] Cancer Genome Atlas Research Network. Electronic address wbe, Cancer Genome Atlas Research N. Comprehensive and integrative genomic characterization of hepatocellular carcinoma. Cell 2017;169(7):1327–41. e1323. 10.1016/j.cell.2017.05.046 PMC5680778 28622513

[88] Shih YL, Kuo CC, Yan MD, Lin YW, Hsieh CB, Hsieh TY. Quantitative methylation analysis reveals distinct association between PAX6 methylation and clinical characteristics with different viral infections in hepatocellular carcinoma. Clin Epigenetics 2016;8:41. 10.1186/s13148-016-0208-3 PMC4841049 27110298

[89] Feng Q, Stern JE, Hawes SE, Lu H, Jiang M, Kiviat NB. DNA methylation changes in normal liver tissues and hepatocellular carcinoma with different viral infection. Exp Mol Pathol 2010;88(2):287–92. 10.1016/j.yexmp.2010.01.002 PMC2848881 20079733

[90] Lambert MP, Paliwal A, Vaissiere T, Chemin I, Zoulim F, Tommasino M, Aberrant DNA methylation distinguishes hepatocellular carcinoma associated with HBV and HCV infection and alcohol intake. J Hepatol 2011;54(4):705–15. 10.1016/j.jhep.2010.07.027 21146512

[91] Tian Y, Arai E, Makiuchi S, Tsuda N, Kuramoto J, Ohara K, Aberrant DNA methylation results in altered gene expression in non-alcoholic steatohepatitis-related hepatocellular carcinomas. J Cancer Res Clin Oncol 2020;146(10):2461–77. 10.1007/s00432-020-03298-4 PMC7467955 32685988

[92] Kuramoto J, Arai E, Tian Y, Funahashi N, Hiramoto M, Nammo T, Genome-wide DNA methylation analysis during non-alcoholic steatohepatitis-related multistage hepatocarcinogenesis: comparison with hepatitis virus-related carcinogenesis. Carcinogenesis 2017;38(3):261–70. 10.1093/carcin/bgx005 PMC5862314 28426876

[93] Dubois F, Bergot E, Zalcman G, Levallet G. RASSF1A, puppeteer of cellular homeostasis, fights tumourigenesis, and metastasis-an updated review. Cell Death Dis 2019;10(12):928. 10.1038/s41419-019-2169-x PMC6895193 31804463

[94] Jakoby WB. The glutathione S-transferases: a group of multifunctional detoxification proteins. Adv Enzymol Relat Areas Mol Biol 1978;46:383–414. 10.1002/9780470122914.ch6 345769

[95] Mercier PL, Bachvarova M, Plante M, Gregoire J, Renaud MC, Ghani K, Characterization of DOK1, a candidate tumour suppressor gene, in epithelial ovarian cancer. Mol Oncol 2011;5(5):438–53. 10.1016/j.molonc.2011.07.003 PMC5528302 21856257

[96] Zhong S, Tang MW, Yeo W, Liu C, Lo YM, Johnson PJ. Silencing of GSTP1 gene by CpG island DNA hypermethylation in HBV-associated hepatocellular carcinomas. Clin Cancer Res 2002;8(4):1087–92. 11948118

[97] Xie Q, Chen L, Shan X, Shan X, Tang J, Zhou F, Epigenetic silencing of SFRP1 and SFRP5 by hepatitis B virus X protein enhances hepatoma cell tumourigenicity through Wnt signaling pathway. Int J Cancer 2014;135(3):635–46. 10.1002/ijc.28697 24374650

[98] Su PF, Lee TC, Lin PJ, Lee PH, Jeng YM, Chen CH, Differential DNA methylation associated with hepatitis B virus infection in hepatocellular carcinoma. Int J Cancer 2007;121(6):1257–64. 10.1002/ijc.22849 17534893

[99] Nieto MA, Huang RY, Jackson RA, Thiery JP. Emt: 2016. Cell 2016;166(1):21–45. 10.1016/j.cell.2016.06.028 27368099

[100] Bouchard MJ, Schneider RJ. The enigmatic X gene of hepatitis B virus. J Virol 2004;78(23):12725–34. 10.1128/JVI.78.23.12725-12734.2004 PMC524990 15542625

[101] Yan Y, Huang P, Mao K, He C, Xu Q, Zhang M, Anti-oncogene PTPN13 inactivation by hepatitis B virus X protein counteracts IGF2BP1 to promote hepatocellular carcinoma progression. Oncogene 2021;40(1):28–45. 10.1038/s41388-020-01498-3 PMC7790756 33051595

[102] Park IY, Sohn BH, Yu E, Suh DJ, Chung YH, Lee JH, Aberrant epigenetic modifications in hepatocarcinogenesis induced by hepatitis B virus X protein. Gastroenterology 2007;132(4):1476–94. 10.1053/j.gastro.2007.01.034 17408664

[103] Qiu X, Zhang L, Lu S, Song Y, Lao Y, Hu J, Upregulation of DNMT1 mediated by HBx suppresses RASSF1A expression independent of DNA methylation. Oncol Rep 2014;31(1):202–8. 10.3892/or.2013.2848 24247422

[104] Niu D, Zhang J, Ren Y, Feng H, Chen WN. HBx genotype D represses GSTP1 expression and increases the oxidative level and apoptosis in HepG2 cells. Mol Oncol 2009;3(1):67–76. 10.1016/j.molonc.2008.10.002 PMC5527876 19383368

[105] Lee H, Sohn JB, Kim SS, Jang KL. DNA methyltransferase inhibitor assay system based on the HBx-induced DNA methylation of E-cadherin. Biochem Biophys Res Commun 2013;438(3):540–5. 10.1016/j.bbrc.2013.07.094 23906757

[106] Zhao Y, Xue F, Sun J, Guo S, Zhang H, Qiu B, Genome-wide methylation profiling of the different stages of hepatitis B virus-related hepatocellular carcinoma development in plasma cell-free DNA reveals potential biomarkers for early detection and high-risk monitoring of hepatocellular carcinoma. Clin Epigenetics 2014;6(1):30. 10.1186/1868-7083-6-30 PMC4391300 25859288

[107] Hu N, Fan XP, Fan YC, Chen LY, Qiao CY, Han LY, Hypomethylated Ubiquitin-Conjugating Enzyme2 Q1 (UBE2Q1) gene promoter in the serum is a promising biomarker for hepatitis B virus-associated hepatocellular carcinoma. Tohoku J Exp Med 2017;242(2):93–100. 10.1620/tjem.242.93 28592717

[108] Siddiqui NN, Ul Haq A, Siddiqui OA, Khan R. DNA methyltransferase 1, 3a, and 3b expression in hepatitis C associated human hepatocellular carcinoma and their clinicopathological association. Tumour Biol 2016;37(8):10487–97. 10.1007/s13277-016-4941-1 26850594

[109] Hassouna MM, Naguib M, Radwan EM, Abdel-Samiee M, Estaphan S, Abdelsameea E. DNA methyltransferases as potential biomarkers for HCV related hepatocellular carcinoma. Asian Pac J Cancer Prev 2020;21(11):3357–63. 10.31557/APJCP.2020.21.11.3357 PMC8033121 33247696

[110] Formeister EJ, Tsuchiya M, Fujii H, Shpyleva S, Pogribny IP, Rusyn I. Comparative analysis of promoter methylation and gene expression end-points between tumourous and non-tumourous tissues from HCV-positive patients with hepatocellular carcinoma. Mutat Res 2010;692(1–2):26–33. 10.1016/j.mrfmmm.2010.07.013 PMC2948626 20736025

[111] Nishida N, Nagasaka T, Nishimura T, Ikai I, Boland CR, Goel A. Aberrant methylation of multiple tumour suppressor genes in aging liver, chronic hepatitis, and hepatocellular carcinoma. Hepatology 2008;47(3):908–18. 10.1002/hep.22110 PMC2865182 18161048

[112] Umer M, Qureshi SA, Hashmi ZY, Raza A, Ahmad J, Rahman M, Promoter hypermethylation of Wnt pathway inhibitors in hepatitis C virus-induced multistep hepatocarcinogenesis. Virol J 2014;11:117. 10.1186/1743-422X-11-117 PMC4076499 24947038

[113] Quan H, Zhou F, Nie D, Chen Q, Cai X, Shan X, Hepatitis C virus core protein epigenetically silences SFRP1 and enhances HCC aggressiveness by inducing epithelial-mesenchymal transition. Oncogene 2014;33(22):2826–35. 10.1038/onc.2013.225 23770846

[114] Lee H, Woo YJ, Kim SS, Kim SH, Park BJ, Choi D, Hepatitis C virus core protein overcomes all-trans retinoic acid-induced cell growth arrest by inhibiting retinoic acid receptor-beta2 expression via DNA methylation. Cancer Lett 2013;335(2):372–9. 10.1016/j.canlet.2013.02.057 23474497

[115] Ripoli M, Barbano R, Balsamo T, Piccoli C, Brunetti V, Coco M, Hypermethylated levels of E-cadherin promoter in Huh-7 cells expressing the HCV core protein. Virus Res 2011;160(1–2):74–81. 10.1016/j.virusres.2011.05.014 21640770

[116] Piciocchi M, Cardin R, Vitale A, Vanin V, Giacomin A, Pozzan C, Circulating free DNA in the progression of liver damage to hepatocellular carcinoma. Hepatol Int 2013;7(4):1050–7. 10.1007/s12072-013-9481-9 26202034

[117] Tokuhisa Y, Iizuka N, Sakaida I, Moribe T, Fujita N, Miura T, Circulating cell-free DNA as a predictive marker for distant metastasis of hepatitis C virus-related hepatocellular carcinoma. Br J Cancer 2007;97(10):1399–403. 10.1038/sj.bjc.6604034 PMC2360234 17940509

[118] Ahmed R, Salama H, Fouad A, Sabry D, AbdAlah el S, Kamal M. Detection of aberrant p16INK4A methylation in sera of patients with HCV-related liver diseases: an Egyptian study. Med Sci Monit 2010;16(9). CR410–415. 20802412

[119] Wang XD. Alcohol, vitamin A, and cancer. Alcohol 2005;35(3):251–8. 10.1016/j.alcohol.2005.04.006 16054987

[120] Udali S, Guarini P, Ruzzenente A, Ferrarini A, Guglielmi A, Lotto V, DNA methylation and gene expression profiles show novel regulatory pathways in hepatocellular carcinoma. Clin Epigenetics 2015;7:43. 10.1186/s13148-015-0077-1 PMC4419480 25945129

[121] Murphy SK, Yang H, Moylan CA, Pang H, Dellinger A, Abdelmalek MF, Relationship between methylome and transcriptome in patients with non-alcoholic fatty liver disease. Gastroenterology 2013;145(5):1076–87. 10.1053/j.gastro.2013.07.047 PMC3805742 23916847

[122] Borowa-Mazgaj B, de Conti A, Tryndyak V, Steward CR, Jimenez L, Melnyk S, Gene expression and DNA methylation alterations in the glycine N-methyltransferase gene in diet-induced non-alcoholic fatty liver disease-associated carcinogenesis. Toxicol Sci 2019;170(2):273–82. 10.1093/toxsci/kfz110 PMC6934890 31086990

[123] Huidobro C, Torano EG, Fernandez AF, Urdinguio RG, Rodriguez RM, Ferrero C, A DNA methylation signature associated with the epigenetic repression of glycine N-methyltransferase in human hepatocellular carcinoma. J Mol Med 2013;91(8):939–50. 10.1007/s00109-013-1010-8 23475283

[124] Dreval K, Tryndyak V, de Conti A, Beland FA, Pogribny IP. Gene expression and DNA methylation alterations during non-alcoholic steatohepatitis-associated liver carcinogenesis. Front Genet 2019;10:486. 10.3389/fgene.2019.00486 PMC6549534 31191608

[125] Fiala C, Diamandis EP. Utility of circulating tumour DNA in cancer diagnostics with emphasis on early detection. BMC Med 2018;16(1):166. 10.1186/s12916-018-1157-9 PMC6167864 30285732

